# Temporal Dominance of B.1.1.7 over B.1.354 SARS-CoV-2 Variant: A Hypothesis Based on Areas of Variant Co-Circulation

**DOI:** 10.3390/life11050375

**Published:** 2021-04-22

**Authors:** Evangelia Georgia Kostaki, Ioulia Tseti, Sotirios Tsiodras, George N. Pavlakis, Petros P. Sfikakis, Dimitrios Paraskevis

**Affiliations:** 1 Department of Hygiene, Epidemiology and Medical Statistics, Medical School, National and Kapodistrian University of Athens, 11527 Athens, Greece; ekostakh@med.uoa.gr; 2 Uni-Pharma S.A., 14564 Kifissia, Greece; jtsetis@uni-pharma.gr; 3 4th Department of Internal Medicine, Attikon University Hospital, Medical School, National and Kapodistrian University of Athens, 12462 Haidari, Greece; tsiodras@med.uoa.gr; 4 Human Retrovirus Section, National Cancer Institute, Frederick, MD 21702, USA; george.pavlakis@nih.gov; 5 1st Department of Prop. Internal Medicine, Medical School, National and Kapodistrian University of Athens, 11527 Athens, Greece; psfikakis@med.uoa.gr

**Keywords:** SARS-CoV-2, variants, co-circulation, dominance, vaccines

## Abstract

Some emergent SARS-CoV-2 variants raise concerns due to their altered biological properties. For both B.1.1.7 and B.1351 variants, named as variants of concern (VOC), increased transmissibility was reported, whereas B.1.351 was more resistant to multiple monoclonal antibodies (mAbs), as well as convalescent and vaccination sera. To test this hypothesis, we examined the proportion of VOC over time across different geographic areas where the two VOC, B.1.1.7 and B.1.351, co-circulate. Our comparative analysis was based on the number of SARS-CoV-2 sequences on GISAID database. We report that B.1.1.7 dominates over B.1.351 in geographic areas where both variants co-circulate and the B.1.1.7 was the first variant introduced in the population. The only areas where B.1.351 was detected at higher proportion were South Africa and Mayotte in Africa, where this strain was associated with increased community transmission before the detection of B.1.1.7. The dominance of B.1.1.7 over B.1.351 could be important since B.1.351 was more resistant to certain mAbs, as well as heterologous convalescent and vaccination sera, thus suggesting that it may be transmitted more effectively in people with pre-existing immunity to other VOC. This scenario would lessen the effectiveness of vaccine and urge the need to update them with new strains.

## 1. Introduction

SARS-CoV-2 has caused a devastating pandemic with serious consequences in global health and economy. Since the first characterization of the SARS-CoV-2 genome, a large collection of sequences (>770 K until the middle of March 2021) has been submitted to the GISAID database (http://www.GISAID.org, accessed on 11 March 2021), thus facilitating the monitoring of global dispersal of the virus as well as the identification of lineages and variants termed as variants of interest (VOI) and variants of concern (VOC). According to the WHO, the definition of “SARS-CoV-2 Variant of Interest” (VOI) is an isolate phenotypically changed compared to a reference isolate or has a genome with mutations that lead to amino acid changes associated with established or suspected phenotypic implications and has been identified to cause community transmission/multiple COVID-19 cases/clusters, or has been detected in multiple countries; or is otherwise assessed to be a VOI by WHO in consultation with the WHO SARS-CoV-2 Virus Evolution Working Group [1]. Phenotypic changes include changes in the epidemiology, antigenicity, or virulence or changes that have or potentially have a negative impact on available diagnostics, vaccines, therapeutics, or public health and social measures. WHO will provide guidance on amino acid changes with established or suspected phenotypic implications, and may be informed by a database on key amino acid changes, or as reported in the scientific literature.

A VOI (as defined previously) is named a variant of concern (VOC) if, through a comparative assessment, it has been demonstrated to be associated with: (i) increase in transmissibility or detrimental change in COVID-19 epidemiology; (ii) increase in virulence or change in clinical disease presentation; or decrease in effectiveness of public health and social measures or available diagnostics, vaccines, or therapeutics; or assessed to be a VOC by WHO in consultation with the WHO SARS-CoV-2 Virus Evolution Working Group [1].

**Figure 1:**
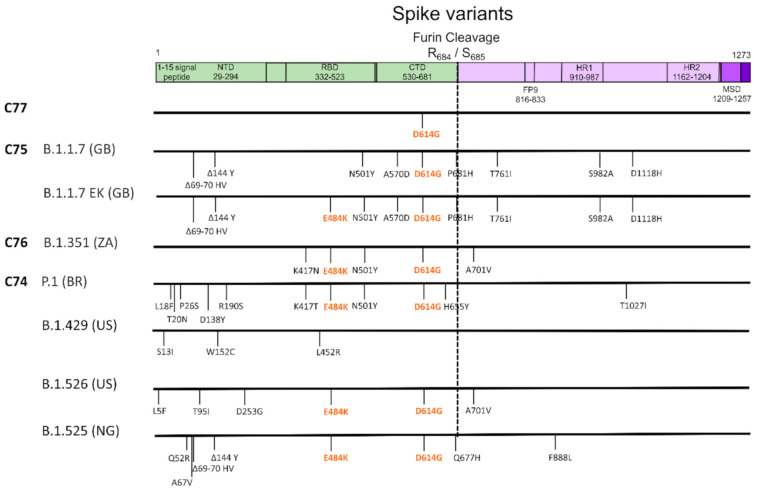
Amino acid mutations and deletions in spike protein for the different variants named after variants of concern (VOC). The different domains of the spike and their length are shown in boxes at the upper part of the figure. Countries are represented by ISO Alpha-2 codes (BR: Brazil, GB: United Kingdom, NG: Nigeria, US: United States, ZA: South Africa).

The earliest interest was focused on the variant harboring the D614G mutation in the spike protein as well as other linked mutations, was reported early on during the course of the pandemic in late January 2020 and was subsequently named C77 (Figure 1). Preliminary experimental evidence was suggestive of increased infectivity compared to the initial virus identified in China [2,3]. In December 2020, the UK authorities announced a new variant named B.1.1.7 or 501Y.V1 responsible for a virus surge across the UK (Figure 1) [4]. At the same time, a second VOC named B.1.351 or 501Y.V2 was announced to rapidly spread in different provinces of South Africa (Figure 1) [5]. The third variant (501Y.V3 or P.1) (Figure 1) was associated with an increased number of cases in Manaus, Brazil [6], a place hit hard by previous pandemic waves with approximately three quarters of its population reportedly developing immunity to SARS-CoV-2. Notably, all these variants embed several amino acid replacements in the spike including some key residues in the receptor binding site (RBD) or other sites. Specifically, all VOC include N501Y in RBD and D614G, while B.1.351 and P.1 have also the E484K in RBD region (Figure 1). B.1.1.7 has additionally two deletions in spike that were unique compared to the other variants. Additional variants with different combinations of mutations have been described such as CAL.20.C (B.1.429), B.1.525 and B.1.526 from southern California [7], Nigeria, and New York [8], respectively, or the B.1.1.7 with the addition of E484K (Figure 1).

These clades have raised concern due to their increased genetic variability and the fact that in preliminary observations they have been associated with virus surges in different geographical areas. Previous studies report that the N501Y mutation in the virus RBD may be associated with increased affinity of the spike protein with the ACE2 receptor in human cells, and therefore with higher transmissibility. In one report, affinity increased by ~3.5-fold [9], whereas, by the addition of the E484K to N501Y, a higher binding affinity (~12.7-fold increase) was documented [10]. In addition, the mean duration of B.1.1.7 associated infection was 13.3 days versus 8.2 days for non-B.1.1.7 viruses. No differences were documented for the peak viral concentrations of 8.5 log10 RNA copies/mL and 8.2 log10 RNA copies/mL for B.1.1.7 and non-B.1.1.7, respectively [11].

Effects on therapeutic efficacy of anti-viral interventions and/or immunization has been the main concern with the emerging variants. A recent study by Wang et al., suggested that B.1.1.7 was refractory to monoclonal antibodies (mAb) against the *N*-terminal of the spike and to a few mAbs to the RBD [12], but maintained susceptibility to convalescent plasma or vaccine induced neutralization. On the other hand, the E484K mutation (present in several emerging variants) has been reported to greatly affect the neutralization activity of monoclonal antibodies and to reduce convalescent plasma or vaccine induced immunity, suggesting that strains with this mutation can probably re-infect people who are vaccinated or have been previously infected with SARS-CoV-2 more easily than other viruses [13,14]. Similar effects have been reported for other mutations in the spike, such as the K417N [12], the K417T in B.1.351 and P.1, and the L18F mutations. The B.1.351 variant is reportedly more refractory to multiple mAbs from several individuals and also more resistant to convalescent plasma and vaccination sera due to the presence of the E484K mutation [12]. This finding was further supported by the diminished efficacy of Novavax Johnson & Johnson and ChAdOx1 vaccines in South Africa where the B.1.351 predominates [15,16,17].

Although the ability to escape pre-existing immunity provides the most reasonable explanation for the fitness advantage of the B.1.351 and P.1 viruses, it does not explain the replicative advantage of B.1.1.7. For this VOC, improved infectivity rather than immune escape appears to contribute to increased fitness and transmission dynamics as detected in the UK, Portugal, and elsewhere.

To the best of our knowledge, potential differences in the replicative advantage between VOC are largely unknown. Given the characteristics and the potential differences in the biological mechanism conferring improved fitness of the VOC, it is important to test the hypothesis of a selective advantage of any of the VOC in settings where the two variants co-circulate. Therefore, we aimed to analyze the proportion of VOC over time in different geographic areas where at least two of the most widely VOC, namely, B.1.1.7 and B.1.351, were present. Selection of these variants was based on the fact that they have been the most widely spread and the ones that co-circulate across different geographic areas.

## 2. Materials and Methods

The analysis was based on the proportions estimated on SARS-CoV-2 sequences available on GISAID database. Specifically, the number of available genomic sequences for B.1.1.7 and B.1.351 was estimated on 11 March 2021, by using the outbreak.info tool (https://outbreak.info, accessed on 11 March 2021). Only countries with more than 50 sequences were included in the analysis. Proportions were estimated using as reference the total number of sequences generated since the first identification of the corresponding variant in each country. The time difference in the date of the first identification of B.1.1.7 versus the B.1.351 was also estimated with positive values corresponding to earlier detection of B.1.1.7.

At the stage of manuscript revision, the number of available genomes belonging to B.1.1.7 and B.1.351 was assessed on 15 April 2021 (https://outbreak.info). Additionally, the number of B.1.1.7 and B.1.351 genomic sequences for different geographic regions was estimated for the time during 15 February and 14 March 2021 based on the data available on GISAID database (https://www.gisaid.org/, accessed on 15 April 2021).

Statistical analysis was performed by fitting a quartile (median) regression model on the difference between the proportions of B.1.1.7 and B.1.351 and the time difference between the earliest variant sequence for B.1.1.7 versus B.1.351. In addition, a multinomial logistic regression model was fitted on 202,005 observations corresponding to sequences from 30 countries published on GISAID database between 15 February and 14 March 2021. We used data only from the last month of our study’s observational period for this model to assure that there was enough time since the B.1.1.7 and B.1.351 introduction to spread in the local population of each country. Variant was defined as the outcome variable on this model, while time difference between the earliest variant sequence for B.1.1.7 versus B.1.351 was chosen as possible explanatory variable. The level of significance was set at 0.05. Both analyses were performed in Stata 13-StataCorp LP software.

## 3. Results

**Figure 2:**
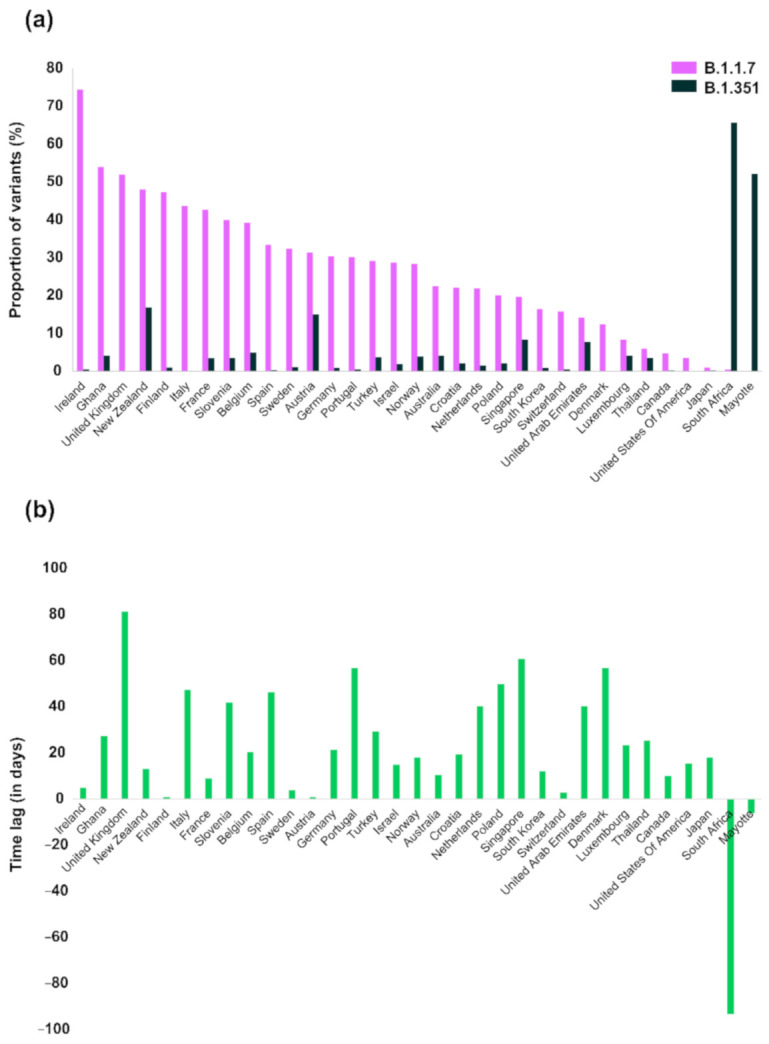
(**a**) Proportion of B.1.1.7 and B.1.351 in different countries based on the number of different genomes available in the GISAID database; (**b**) Time difference between the earliest variant sequence for B.1.1.7 versus the B.1.351 variants of concern (VOC).

Analysis of available data showed that in the vast majority of the countries (31 out of 33), the proportion of B.1.1.7 cases was higher than the corresponding proportion of B.1.351 (Figure 2a). The only areas where B.1.351 dominated were South Africa and Mayotte, an overseas department of France located in the Indian Ocean off the coast of Southeast Africa. South Africa was the place where the B.1.351 was first identified and associated with a virus surge in early December 2020 [5]. Countries where both variants were detected at high proportions were New Zealand, Austria, Singapore, and the United Arab Emirates. New Zealand experienced a first epidemic wave between March and May 2020. Since then, virus spread has been successfully controlled with an approximate number of active cases equal to 93; the 7-day average number of cases ranges between 1 and 11 cases. Similarly, in Singapore the 7-day average number of cases was approximately between 10 and 31 cases after September 2020, suggesting that no virus surges were detected after the introduction of the variants in these countries. In the United Arab Emirates, a virus surge was detected at the beginning of the new year and since February 2021 the number of cases has been declining. The number of sequenced viruses from the United Arab Emirates was limited to 148 and 64 for B1.1.7 and B.1.351, respectively. This is only a small proportion compared to the total number of diagnosed cases in the same time period. In Austria, both variants have been detected and they have been mostly concentrated in the Tyrol state. Although the B.1.1.7 identified first in the UK, where it has been dominating at proportions higher than 75% since the beginning of the current year, it has surpassed B.1.351 across different regions where both variants co-circulate. The proportion of available genomes for the two variants was re-estimated for the time after 29 March 2021 and until 11 April 2021. A similar patten was observed about the dominance of B.1.1.7 versus B.1.251 (Table S1).

To investigate whether these differences were due to the longer presence of B.1.1.7 in remote geographic areas, we plotted the time lag since the first identification of the two strains (earliest sequence available per clade) (Figure 2b). More specifically, the time lag corresponds to the time difference between the date of the earliest variant sequence for each geographic region. The differences were more pronounced for the countries where both variants identified first, i.e., for the UK and South Africa. Except for these areas, the median time difference was 19 days (IQR: 10, 40).

Measurements on the proportion of the B.1.1.7 were available for some countries using different methodologies. Some used as a proxy the S gene target failure (SGTF) in real-time PCR assays, DNA sequencing or a combination of the two methods. The proportions shown in Table 1 were based on the number of cases diagnosed during a period of one week (week 7 until 9 of 2021). The countries were shown according to the proportion of the B.1.1.7 variant in order for the corresponding proportions of the B.1.351 to be comparable. The figures in Table 1, as expected, were different from the cumulative proportions of B.1.1.7 since the first day of the variant identification. For all countries, apart from Poland, the SGTF proportions were higher than the cumulative proportions, suggesting an increasing trend of the B.1.1.7 in the corresponding countries. Similar figures for the B.1.351 were not available.

To further investigate if the difference in the first detection of each variant was associated with the difference between the variants’ proportions, we performed statistical analysis by fitting a quartile (median) regression model to the data. Analysis revealed that for a one day increase in the time difference between the earliest variant sequence for B.1.1.7 versus B.1.351 (time lag), the predicted value of change in the difference between the proportions of B.1.1.7 and B.1.351 increases by 0.4 (95% CI: 0.1–0.8; *p* = 0.021).

Furthermore, the multinomial logistic regression analysis revealed that if the time lag were increased by one day, the relative risk for B.1.1.7 compared to B.1.351 would be expected to increase by a factor of 1.07 (95% CI: 1.02–1.11; *p* = 0.002). Particularly, a one day increase in the time lag increases the relative risk of Β.1.1.7 (compared to Β.1.351) by about 7%.

## 4. Discussion

Our analysis suggests that in countries where both B.1.1.7 and B.1.351 co-circulate, B.1.1.7 has dominated local transmission patterns, except for the South Africa where B.1.351 variant has been associated with community transmission across different provinces before the introduction of B.1.1.7 [5]. Statistical analysis confirmed our findings and specifically the multinomial logistic regression analysis revealed that a one day increase in the time lag increases the relative risk of Β.1.1.7 (compared to Β.1.351) by about 7%. We also found that B.1.1.7 was introduced before the South African lineage in Europe, Asia and the Americas, but in several areas the time difference since the first identification of the two strains was not pronounced. The transmission pattern of the two variants could also be explained due to the limited importation events in Europe from remote South Africa compared to the UK. On the other hand, as soon as B.1.351 has entered Europe as early as 10 December 2020 (Table 1), it could have spread in Europe through internal flights.

The selection advantage of different viruses is a dynamic process depending on the complex interplay between the pathogen and the host suggesting that different strains can dominate in different environments [18,19]. Selection of B.1.1.7 variant and dominance over the pre-existing viruses was probably due to its increased transmissibility rather than its ability to re-infect people with existing immunity [12]; however, this may change in the future when vaccination coverage increases. Selection advantage therefore cannot be determined across different populations and time points since it depends on several factors such as the levels of the existing immunity, the characteristics of the strains for which immunity was developed, climatic factors, the speed of the vaccination programs, compliance to public health measures, as well as other factors, importantly, the virus mutation rate, and its ability to recombine. In this study, we show that, under the conditions of the pandemic since the late 2020 and before we reach high vaccination levels, the B.1.351 virus does not dominate over B.1.1.7 in geographic areas where both variants co-circulate, and the B.1.1.7 was the first variant introduced in the population. However, our findings cannot exclude the risk for the B.1.351 to cause outbreaks due to its ability to escape immunity to other variants.

One major limitation of our study is that the analyzed viral genomes may not be representative of the circulated viruses in each region. Our analysis was based on sequencing data and information on the weekly proportions of the B.1.1.7 which confirmed that this variant was more prevalent than the B.1.351 across different geographic regions; thus, suggesting that B.1.1.7 dominates B.1.351 in areas where both variants co-circulate and the former variant was the first associated with community transmission. Another limitation was that the earliest variant date did not accurately reflect the day zero of the virus introduction in a particular area, but it provides a proxy of that date. Our study suggests that that the B.1.1.7 variant appears to be the only one able to dominate over the B.1.351 variant. Moreover, the minimum proportion or the minimum incidence of the B.1.1.7 variant necessary to keep the B.1.351 variant under control cannot be estimated from the current data.

The dominance of B.1.1.7 over B.1.351 could be of importance since the latter is more resistant to certain mAbs, convalescent plasma or vaccination sera [12], thus suggesting that it may be transmitted more effectively in people with pre-existing immunity. This scenario would lessen the effectiveness of vaccine and urge the need to update them with new strains. Based on the existing data, our study highlights that B.1.351 may have similar fitness or a disadvantage compared to B.1.1.7 in areas where it is associated with community transmission, therefore raising hope that a more efficient “immune-evade” VOC cannot cause a widespread dispersal over B.1.1.7. The future transmission dynamics cannot be easily forecasted, but the current message is that the transmission advantage of B.1.1.7 is probably higher than B.1.351, and this characteristic can protect us from the worldwide dispersal of a more dangerous variant for now. It remains to be seen whether a subsequent epidemic can be triggered by B.1.351 once the B.1.1.7 epidemic has subsided. In addition, the possibility of recombination generating a virus combining the better transmission of B.1.1.7 with the more severe pathologies of B.1.351 has to be considered and may shape the direction of virus evolution. Furthermore, this may create additional opportunities for new virus transmissions.

## Figures and Tables

**Table 1:** Information related to the number of sequences, the earliest variant sequence and the corresponding proportions for B.1.1.7 and B.1.351 variants of concern (VOC).

VOC	B.1.1.7					B.1.351			
Countries	Proportion (%)	Proportion of SGTF ^1^—Week 8 of 2021 (%)	Earliest Variant Sequence	Number of Variant Sequences	Number of Sequences ^2^	Proportion (%)	Earliest Variant Sequence	Number of Variant Sequences	Number of Sequences ^2^
Ireland	74.50	88.6	17/12/2020	1966	2639	0.62	22/12/2020	16	2594
United Kingdom	51.99	NA ^3^	20/09/2020	111,140	213,761	0.16	10/12/2020	212	135,667
Ghana	54.03	NA	10/12/2020	67	124	4.21	06/01/2021	4	95
Finland	47.43	NA	18/12/2020	268	565	1.07	19/12/2020	6	563
Italy	43.67	NA	14/12/2020	2244	5139	0.21	30/01/2021	8	3788
France	42.75	65.8	13/12/2020	1847	4320	3.48	22/12/2020	144	4137
Slovenia	40.00	NA	29/12/2020	46	115	3.64	09/02/2021	2	55
Belgium	39.33	46.3	30/11/2020	2064	5248	5.02	20/12/2020	244	4857
Spain	33.53	25–30	08/11/2020	1323	3946	0.34	24/12/2020	10	2930
Sweden	32.39	41.5 ^4^	20/12/2020	458	1414	1.22	24/12/2020	16	1310
New Zealand	48.09	NA	16/12/2020	63	131	16.96	29/12/2020	19	112
Portugal	30.17	50.5	09/11/2020	746	2473	0.51	04/01/2021	9	1782
Germany	30.44	54.5	30/11/2020	4427	14,543	1.03	21/12/2020	143	13,817
Israel	28.74	~90 ^5^	16/12/2020	434	1510	1.92	31/12/2020	16	833
Turkey	29.15	NA	24/12/2020	479	1643	3.78	22/01/2021	54	1428
Norway	28.49	72.5	09/12/2020	337	1183	3.92	27/12/2020	40	1021
Netherlands	22.00	64.3 ^5^	12/11/2020	1697	7715	1.63	22/12/2020	99	6091
Croatia	22.06	NA	20/01/2021	60	272	2.13	09/02/2021	2	94
Australia	22.52	NA	30/11/2020	134	595	4.14	10/12/2020	23	556
Poland	20.08	9	22/12/2020	151	752	2.11	10/02/2021	2	95
Austria	31.49	63.2	22/12/2020	336	1067	15.09	23/12/2020	159	1054
South Korea	16.45	NA	14/12/2020	90	547	0.97	26/12/2020	4	413
Switzerland	15.80	40.5 ^5^	09/11/2020	1983	12,550	0.63	12/11/2020	77	12,313
Denmark	12.47	76.5	09/11/2020	4889	39,191	0.06	04/01/2021	12	19,655
Singapore	19.70	NA	08/12/2020	66	335	8.45	07/02/2021	6	71
United Arab Emirates	14.19	NA	16/11/2020	21	148	7.81	26/12/2020	5	64
Canada	4.83	NA	15/12/2020	54	1117	0.26	25/12/2020	2	761
Luxembourg	8.40	65.5	24/12/2020	32	381	4.17	16/01/2021	2	48
United States of America	3.58	26.2	17/12/2020	2652	74,129	0.06	01/01/2021	38	62,390
Thailand	6.06	NA	08/01/2021	8	132	3.61	03/02/2021	3	83
Japan	1.07	NA	01/12/2020	59	5527	0.26	19/12/2020	9	3441
Mayotte	0.19	NA	13/01/2021	1	518	52.24	07/01/2021	338	647
South Africa	0.47	NA	09/01/2021	1	213	65.74	08/10/2020	1086	1652

^1^ SGTF: S gene target failure. ^2^ This number corresponds to the total number of characterized sequences irrespective of their classification since the identification of the corresponding variant. The numbers are different for the two variants due to the difference in dates of earliest variant sequence. ^3^ NA: Not available. ^4^ Based on sequencing of all variants with N501Y + A570D. ^5^ Data available for week 7.

## Data Availability

Publicly available data were downloaded from www.gisaid.org (accessed on 11 March 2021).

## Supplementary Materials

The following are available online at https://www.mdpi.com/article/10.3390/life11050375/s1, Table S1: Information related to the number of variant sequences, the earliest variant sequence, the last detection date, and the corresponding proportions for B.1.1.7 and B.1.351 variants of concern (VOC).
