# Supplementary material for: Temporal Dominance of B.1.1.7 over B.1.354 SARS-CoV-2 Variant: A Hypothesis Based on Areas of Variant Co-Circulation
Source: Life (Basel). 2021 Apr 22;11(5):375. doi: 10.3390/life11050375 (PMC8143446; doi:10.3390/life11050375)
Supplement: Supplementary file 1 [file life-11-00375-s001.zip › life-1192416-supplementary.pdf]

Table S1. Information related to the number of variant sequences, the earliest variant sequence, the last detection date, and the corresponding proportions for B.1.1.7 and B.1.351 variants of concern (VOC).

VOC	B.1.1.7						B.1.351				
**Countries**	**Proportion (%)**	**Proportion of SGTF[1] - week 8 of 2021 (%)**	**Earliest variant sequence**	**Number of variant sequences (updated)[2]**	**Proportion (updated) (%)[2]**	**Date last detected**	**Proportion (%)**	**Earliest variant sequence**	**Number of variant sequences (updated)[2]**	**Proportion (updated) (%)[2]**	**Date last detected**
Ireland	74.50	88.6	17/12/2020	6394	71.1	02/04/2021	0.62	22/12/2020	47	0.5	02/04/2021
United Kingdom	51.99	NA[3]	20/09/2020	200299	54.5	09/04/2021	0.16	10/12/2020	501	0.1	09/04/2021
Ghana	54.03	NA	10/12/2020	NA	NA	NA	4.21	06/01/2021	NA	NA	NA
Finland	47.43	NA	18/12/2020	NA	NA	NA	1.07	19/12/2020	NA	NA	NA
Italy	43.67	NA	14/12/2020	9574	51.9	09/04/2021	0.21	30/01/2021	49	0.3	09/04/2021
France	42.75	65.8	13/12/2020	7511	43.6	08/04/2021	3.48	22/12/2020	620	3.6	08/04/2021
Slovenia	40.00	NA	29/12/2020	900	15.9	31/03/2021	3.64	09/02/2021	26	0.5	31/03/2021
Belgium	39.33	46.3	30/11/2020	6352	43.9	07/04/2021	5.02	20/12/2020	691	4.8	07/04/2021
Spain	33.53	25-30	08/11/2020	5185	28.3	07/04/2021	0.34	24/12/2020	38	0.2	07/04/2021
Sweden	32.39	41.5[4]	20/12/2020	4471	35.5	05/04/2021	1.22	24/12/2020	300	2.4	05/04/2021
New Zealand	48.09	NA	16/12/2020	98	9.8	30/03/2021	16.96	29/12/2020	23	2.3	30/03/2021
Portugal	30.17	50.5	09/11/2020	NA	NA	NA	0.51	04/01/2021	NA	NA	NA
Germany	30.44	54.5	30/11/2020	28893	52.0	07/04/2021	1.03	21/12/2020	897	1.6	07/04/2021
Israel	28.74	~90[5]	16/12/2020	NA	NA	NA	1.92	31/12/2020	NA	NA	NA
Turkey	29.15	NA	24/12/2020	528	15.3	29/03/2021	3.78	22/01/2021	124	3.6	29/03/2021
Norway	28.49	72.5	09/12/2020	NA	NA	NA	3.92	27/12/2020	NA	NA	NA
Netherlands	22.00	64.3[5]	12/11/2020	8403	40.5	04/04/2021	1.63	22/12/2020	383	1.8	04/04/2021
Croatia	22.06	NA	20/01/2021	NA	NA	NA	2.13	09/02/2021	NA	NA	NA
Australia	22.52	NA	30/11/2020	252	1.5	11/04/2021	4.14	10/12/2020	41	0.2	11/04/2021
Poland	20.08	9	22/12/2020	3402	65.2	04/04/2021	2.11	10/02/2021	14	0.3	04/04/2021
Austria	31.49	63.2	22/12/2020	1044	28.5	29/03/2021	15.09	23/12/2020	235	6.4	29/03/2021
South Korea	16.45	NA	14/12/2020	104	3.2	05/04/2021	0.97	26/12/2020	5	0.2	05/04/2021
Switzerland	15.80	40.5[5]	09/11/2020	5859	22.7	06/04/2021	0.63	12/11/2020	137	0.5	06/04/2021

Denmark	12.47	76.5	09/11/2020	NA	NA	NA	0.06	04/01/2021	NA	NA	NA
Singapore	19.70	NA	08/12/2020	126	6.1	07/04/2021	8.45	07/02/2021	118	5.7	07/04/2021
United Arab Emirates	14.19	NA	16/11/2020	NA	NA	NA	7.81	26/12/2020	NA	NA	NA
Canada	4.83	NA	15/12/2020	3115	11.3	30/03/2021	0.26	25/12/2020	88	0.3	30/03/2021
Luxembourg	8.40	65.5	24/12/2020	1467	24.7	30/03/2021	4.17	16/01/2021	415	7.0	30/03/2021
United States of America	3.58	26.2	17/12/2020	31421	11.4	09/04/2021	0.06	01/01/2021	513	0.2	09/04/2021
Thailand	6.06	NA	08/01/2021	44	4.9	06/04/2021	3.61	03/02/2021	7	0.8	06/04/2021
Japan	1.07	NA	01/12/2020	455	1.5	07/04/2021	0.26	19/12/2020	22	0.1	07/04/2021
Mayotte	0.19	NA	13/01/2021	NA	NA	NA	52.24	07/01/2021	NA	NA	NA
South Africa	0.47	NA	09/01/2021	NA	NA	NA	65.74	08/10/2020	NA	NA	NA

[1] SGTF: S gene target failure, [2] The number of variant sequences and proportions were updated to the corresponding last detection date that is later than 29 March 2021, [3] NA: Not available, [4] Based on sequencing of all variants with N501Y+A570D, [5] Data available for week 7
